# A Scoping Review of the Lived Experiences of Individuals With Huntington's Disease, Their Informal Caregivers and Offspring

**DOI:** 10.1111/jan.17024

**Published:** 2025-05-08

**Authors:** Cathelijn Van Baar, Kristel Kalkers, Sascha Bolt, Raymund Roos, Robbert Gobbens

**Affiliations:** ^1^ Tranzo, Tilburg School of Social and Behavioral Sciences Tilburg University Tilburg the Netherlands; ^2^ Center of Expertise Huntington, Mijzo Raamsdonksveer the Netherlands; ^3^ Department of Neurology Leiden University Medical Center Leiden the Netherlands; ^4^ Faculty of Health, Sports and Social Work Inholland University of Applied Sciences Amsterdam the Netherlands; ^5^ Zonnehuisgroep Amstelland Amstelveen the Netherlands

**Keywords:** barriers, caregiver, family, Huntington's disease, lived experiences, nursing

## Abstract

**Background:**

Huntington's disease has a disruptive effect on entire families. While previous reviews have examined lived experiences of individuals with Huntington's Disease, their informal caregivers, or their offspring, none have provided a comprehensive overview that integrates these three perspectives.

**Design:**

A scoping review.

**Aims:**

Providing an integrated view and a holistic understanding of the multifaceted challenges faced by families affected by Huntington's disease.

**Methods:**

We operationalised the lived experiences using the keywords: “barriers”, “facilitators” and “needs”. We extracted and thematically analysed data from 35 articles searched from 1993 to 2023.

**Results:**

Twelve themes were identified, organised into three dimensions: (1) *Having the Disease*: encompassing the symptoms and progression of the disease; (2) *Family Dynamics:* reflecting the challenges of living in a household affected by Huntington's disease; and (3) *Outside World*: describing relationships and interactions with relatives, friends, health services and wider social structures.

**Conclusions:**

The dimensions and related experiences of all three perspectives are intertwined. These experiences are mutually reinforcing, with fluid shifts in perspective occurring between family members. While the needs of family members overlap, they also diverge, highlighting the need for a systemic, family‐centred approach to address the evolving challenges faced by all family members.

**Patient or Public Contribution:**

No Patient or Public Contribution.

**Summary**

- What does this paper contribute to the wider global clinical community?
  ○ It provides a holistic understanding of the impact of the disease on families.
  ○ It serves as a broader illustration of how chronic genetic diseases can reshape family dynamics, social relations, and community interactions.

## 1 Introduction

Huntington's disease (HD) is a rare progressive neurodegenerative autosomal dominant inherited disorder that is incurable (Pringsheim et al. 2012).

HD is characterised by involuntary choreatic movements, and by behavioural, psychiatric and cognitive disorders (Roos 2010). Individuals with HD may experience a constellation of deteriorating issues, including short‐term memory deficit, mood swings or depression, aggression, dysphagia, and challenges with mobility and communication (Domaradzki 2015). Typically, the age at onset of HD is between 30 and 50 years (Parekh et al. 2018); the average duration from the manifestation of HD symptoms to death is 17 to 20 years (Roos 2010). An HD diagnosis is made based on clinical symptoms in individuals with an affected parent and is confirmed by DNA testing. Adults who are at risk may undergo pre‐manifest testing to determine their mutation status (Roos 2010). HD prevalence, more precisely the prevalence of diagnosis, varies more than tenfold between different geographical regions, with the highest rates in the Western countries (3.6–7.3 per 100,000) and significantly lower rates in Asia (0.40 per 100,000) and Africa (no data). This variation is linked to genetic founder effects and migration patterns, with a higher concentration of HD mutations in populations of European ancestry. In Africa, underdiagnosis due to limited healthcare access may also play a role in these lower rates (Rawlins et al. 2016).

Current treatment of HD focuses on symptom management to enhance or maintain quality of life for as long as possible (Parekh et al. 2018). Individuals with HD might gain from a coordinated multidisciplinary approach to healthcare, which includes ongoing monitoring, neurological and cognitive evaluations, as well as physiotherapy, speech therapy, and occupational therapy (Frich et al. 2016). The progressive nature of HD presents significant barriers to maintaining independence. Over time, individuals with HD require 24‐h care and help with all activities of daily living (Parekh et al. 2018). While specialised HD care centres offer multidisciplinary support, the availability and quality of services vary by region (Frich et al. 2016).

As the disease progresses, the quality of life of individuals with HD and their informal caregivers declines (Exuzides et al. 2022; Van Walsem et al. 2017). Frequently reported aspects of burden include the impact of changing roles within families, social isolation, neglected needs, negative impact on the family system, and an experienced general lack of support (Aubeeluck et al. 2012; Domaradzki 2015).

Although there are similarities between caregiving for someone with HD and caring for relatives with other neurodegenerative conditions, such as Alzheimer's or Parkinson's disease, HD presents several unique challenges. These challenges arise from the hereditary nature of HD (children have a 50% chance of inheriting the disease), the wide variety of signs and symptoms, the young age at onset (when individuals still have young families and careers), the duration, and the fact that it is a rare disease (Daemen et al. 2023). Consequently, there is a lack of recognition and understanding within the healthcare system and society (Domaradzki 2015; Parekh et al. 2018). Due to the combination of these unique challenges, informal caregivers of individuals with HD report emotional distress, anticipatory grief, loss of self‐care concerns about their children's risk of developing HD, and difficulties with the unpredictable behaviour of the individual with HD (Parekh et al. 2018).

In addition to individuals with HD and their informal caregivers, children who grow up in families affected by HD also bear a significant burden (Cooper et al. 2025). Children who have taken on a caregiving role of a parent with HD describe feelings of strain, isolation, disruption from school, and a lack of support due to the caregiving burden, the invisibility of their parent's condition, and their caregiving role to others (Kavanaugh et al. 2015). Knowing that they are at risk of eventually developing HD themselves often worsens the caring experience, even more so when they are confirmed HD mutation carriers (Kavanaugh et al. 2015).

Overall, these distinctive aspects of HD underscore the disruptive impact of the disease on the entire family (Parekh et al. 2018). To gain insight into this impact, it is valuable to better understand the lived experiences of families affected by HD. Lived experiences refer to a person's experiential knowledge gained from direct involvement in a specific situation, interaction, or event (Given 2008). Previous reviews have examined lived experiences with HD separately from the perspectives of affected individuals (Mahmood et al. 2022), informal caregivers (Domaradzki 2015; Parekh et al. 2018), and offspring (Cooper et al. 2025). An integrated view that combines these three perspectives to provide a holistic understanding of the multifaceted challenges faced by families affected by HD is lacking. The current review aims to fill this knowledge gap.

## 2 The Review

### 2.1 Aim

The aim of this scoping review is to provide an integrated view and a holistic understanding of the multifaceted challenges faced by families affected by HD. It will map and integrate the lived experiences from three perspectives: (1) individuals with HD, (2) their informal caregiver, and (3) their offspring.

### 2.2 Design

Scoping review methodology is suitable for addressing the broad range and exploratory nature of the current research aim (Tricco et al. 2018). This scoping review was conducted using the Joanna Briggs Institute methodology and reported according to the Preferred Reporting Items for Systematic Reviews and Meta‐Analyses for Scoping Reviews (PRISMA‐ScR) guidelines (Tricco et al. 2018).

### 2.3 Search Methods

The databases Medline, PsycINFO, Cinahl, Embase and Google Scholar were searched for papers published up to August 2023. The keywords were identified from nine preliminary articles about the lived experiences of HD from the perspective of individuals with HD, their informal caregivers and their offspring. This resulted in the identification of key search terms—“barriers”, “facilitators”, and “needs”—to capture the lived experiences in everyday life with HD. All nine preliminary articles were successfully retrieved by the final search. The reference lists of other relevant reviews (Domaradzki 2015; Mahmood et al. 2022; Parekh et al. 2018) were hand‐searched to identify any additional literature. The search string was tailored for each database. The Medline search string is included in the Appendix for reference.

### 2.4 Inclusion and Exclusion Criteria

Only original qualitative studies published in English since 1993, the year the HD gene was discovered, were included. Mixed methods studies were eligible as long as the qualitative data could be clearly distinguished. The population of the included studies had to be individuals with HD living at home, their informal caregivers and/or their offspring living in the same household. The focus of the studies had to be on the barriers, facilitators and needs in everyday living with HD. Letters, review articles, evaluations of interventions and articles describing quantitative measures of clinical symptoms of HD, without a link to lived experiences, were excluded. Studies that focused only on the impact of DNA testing, juvenile HD and end‐stage disease were also excluded.

### 2.5 Search Outcome

**FIGURE 1:**
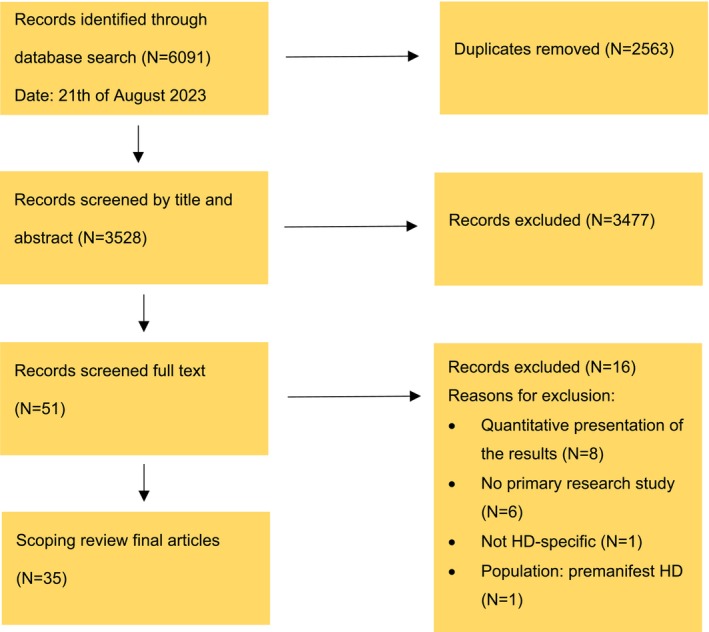
Prisma‐screening flow diagram.

The initial literature search identified 6091 articles. After removing duplicates, 3528 unique articles remained. 400 articles were double‐checked for title and abstract by two authors (C.B. and either K.K. or S.B.). Differences were discussed between the three authors and the exclusion criteria were refined. The titles and abstracts of the remaining articles were screened for relevance by the first author. A second author (K.K.) was consulted in case of uncertainty about inclusion. Of the remaining 51 research articles, 18 were read in full and assessed for eligibility by the three authors independently. Any disagreements were discussed between them to reach consensus. The remaining 33 articles were read in full by the first author. This resulted in the inclusion of 35 articles (see Figure 1).

### 2.6 Quality Appraisal

As inclusion was not based on quality, no quality assessment was carried out. The aim was to include the available studies that met the defined criteria.

### 2.7 Data Abstraction

A predefined extraction form was used to extract data related to lived experience. Initially, 10 randomly selected articles were extracted by two authors (C.B. and K.K.) independently to test the extraction form. Three authors (C.B., K.K. and S.B.) reviewed these extracts for similarities and differences. Differences were discussed and resulted in the final extraction form. The remaining 25 articles were then extracted by the first author.

### 2.8 Synthesis

Extracts from the included studies were analysed using an inductive thematic approach. To make the data manageable and to support thematisation, the final extracts were open coded in Atlas.ti by the first author. Ten percent of the included articles were also coded by a second author (K.K.) to identify differences and similarities and to increase trustworthiness. Through axial coding, which distinguishes between main and sub‐codes, open codes were clustered into higher order categories, resulting in 12 themes. Three authors (C.B., K.K. and S.B.) discussed these themes to ensure that they were complete and logically arranged and to reach a mutual understanding of the content of these themes. The lived experiences described in these 12 themes were checked for completeness by two authors (C.B. and K.K.). To provide an organised and structured way of presenting the experiences of all family members, these 12 themes were grouped into three dimensions.

## 3 Results

### 3.1 Study Characteristics

The 35 studies were focused on the lived experiences of individuals with HD (*n* = 18), the informal caregivers (*n* = 19) and/or the offspring (*n* = 10). Ten studies reported from two or three perspectives. All studies were conducted in Western countries. Most studies used semi‐structured interviews (*n* = 21) or focus groups (*n* = 8). The studies were published between 2004 and 2023. The study characteristics of the 35 included articles are presented in Table 1 and their objectives in Table 2.

**TABLE 1:** Characteristics of the included articles (*n* = 35).

	*N*
Population of the studies	
Individuals with HD and their informal caregivers	9
Informal caregivers	9
Offspring of individuals with HD	8
Individuals with HD	7
Individuals with HD and their offspring	1
Individuals with HD, their informal caregivers and their offspring	1
Concept of the studies	
Impact of caregiving for an individual with HD	10
Growing up in a HD‐affected family	5
End of life wishes of the individual with HD	3
Communication skills of the individual with HD	3
Needs and concerns in living with HD of individuals with HD and informal caregivers	3
Psychosocial functioning of individuals with HD and family members	2
Quality of life of individuals with HD or informal caregivers	2
Healthcare needs of individuals with HD and informal caregivers	2
Talking about HD in the family	1
Impact of chorea on everyday functioning of individuals with HD	1
Impact of apathy on physical activity of individuals with HD	1
Depression in individuals with HD	1
Views of individuals with HD on their future	1
Geographical focus	
United States of America (USA)	8
Norway	5
United Kingdom (UK)	4
USA & Canada	3
Netherlands	3
Australia	3
Scotland	2
Canada	2
USA & UK	1
German	1
Sweden	1
Slovakia	1
Malta	1
Study design	
Semi‐structured interview	21
Focus group	8
Combined semi‐structured interview and focus group	2
Written questionary including narrative comments	2
Mixed method	2

**TABLE 2:** Objectives of the included articles.

First author and year		Population	Study objectives
1	Atkins et al. 2022	I	Examine the experience of apathy of individuals with HD and its impact on physical activity
2	Aubeeluck et al. 2012	C	Explore the quality of life (QOL) of informal caregivers of individuals with HD
3	Bilal et al. 2022	I	Examine the lived experience of depression in individuals with CAG expansion for HD
4	Booij et al. 2013	I	Examine the thoughts and attitudes of individuals with HD to euthanasia, physician‐assisted suicide and use of advance directives
5	Carlozzi and Tulsky 2013	I and C	Explore the health‐related QOL (HRQOL) of individuals with HD
6	Daemen et al. 2023	C	Gain insight into the barriers and facilitators of caring for individuals with HD and identify needs of informal caregivers for support through a remote programme
7	Dawson et al. 2004	I and C	Explore the needs for palliative care service provision of individuals with HD and their families
8	Dondanville et al. 2019	O	Explore the interaction between offspring's perception of genetic risk for HD, caregiving experiences and thoughts about predictive testing
9	Ekkel et al. 2021	I	Gain insight into the views of individuals with HD about their future and how they cope with the poor prognosis of HD
10	Etchegary 2011	C	Explore the healthcare experiences and suggestions for improvement of families affected by HD
11	Forrest Keenan et al. 2007	O	Describe the experiences of offspring growing up in a family affected by HD
12	Grimstvedt et al. 2021	I	Examine the communication‐related experiences of individuals with HD and their professional caregivers
13	Halpin 2012	I and C	Explore whether suicide in individuals with HD is due to mental pathology or rational decision
14	Hartelius et al. 2010	I and C	Explore how communication is affected by HD
15	Ho and Hocaoglu 2011	I	Explore what individuals with HD are most concerned about at different stages of HD
16	Hubcikova et al. 2022	I and C	Explore the socio‐psychological impact of HD on individuals with HD and informal caregivers and incentives to improve healthcare
17	Kavanaugh et al. 2015	O	Explore the needs of offspring (age 12–20) of parents with HD
18	Kavanaugh et al. 2016	O	Explore the offspring's knowledge of end of life (EOL) wishes of the parent with HD and their willingness to have EOL discussions
19	Kjoelaas, Feragen, et al. 2022; Kjoelaas, Jensen, et al. 2022	O	Explore the current and past experiences of social support for offspring who are growing up with a parent with HD
20	Kjoelaas, Feragen, et al. 2022; Kjoelaas, Jensen, et al. 2022	C and O	Gain insight into how offspring and informal caregivers experience talking about HD in childhood
21	Lowit and van Teijlingen 2005	C	Gain an in‐depth understanding of the caregiver role for individuals with HD
22	Mand et al. 2015	O	Explore the psychosocial context of experience and specific challenges faced by offspring living in families affected by HD
23	Maxted et al. 2014	I, C and O	Examine the dyadic experience of psychosocial functioning in families affected by HD
24	Rothing et al. 2014	C	Explore the informal caregivers' experience with the impact of HD on family structures and roles
25	Rothing et al. 2015	C	Explore the coping strategies and behaviours of informal caregivers to care for themselves, while caring for individuals with HD
26	Scerri 2015	I and C	Explore the experiences of Maltese informal caregivers caring for an individual with HD
27	Sherman et al. 2020	I and C	Explore the impact of chorea on everyday functioning and identify patterns of perception and experience of chorea among individuals with HD, informal caregivers and professionals
28	Smith et al. 2015	I and C	Identify what is most helpful in living with HD from the perspective of individuals with HD, informal caregivers and professionals
29	Soltysiak et al. 2008	I and C	Identify the needs and coping strategies of individuals with HD and their family members
30	Sparbel et al. 2008	O	Explore the experience of offspring living in families affected by HD
31	Velissaris et al. 2021	C	Identify the psycho‐educational and emotional support needs of male caregivers of spouses with HD
32	Williams, Ayres, et al. 2009; Williams, Skirton, et al. 2009	O	Describe the caregiving experience of offspring of individuals with HD
33	Williams et al. 2012	C	Examine and compare the personal concerns of informal caregivers of individuals with HD in UK and USA
34	Williams, Ayres, et al. 2009; Williams, Skirton, et al. 2009	C	Examine and describe the emotional experience of caring and coping strategies of informal caregivers of individuals with HD
35	Zarotti et al. 2019	I	Explore the perspective of individuals with HD on their own communication skills

Abbreviations: C, informal caregiver; I, individual with HD; O, offspring.

### 3.2 Daily Living With HD: A Three‐Dimensional Perspective

The analysis resulted in 12 themes, which were grouped in the following dimensions (see Table 3):

- Having the Disease: This dimension shows the physical and cognitive symptoms, behavioural changes, and disease progression related to HD, as experienced by individuals with HD, their informal caregivers, and their offspring.
- Family Dynamics: This dimension presents the experiences of the three perspectives that focus on living in a household affected by HD.
- Outside World: This dimension provides an overview of the experiences of the three perspectives in relationships and interactions with relatives, friends, school, work, health services, and society.

**TABLE 3:** The 12 themes grouped in three dimensions.

Dimensions	Themes
Having the disease	Navigating the diverse symptoms of HD
	2 Fading capacities: the toll of disease progression
B Family dynamics	3 Hidden conversations and strained relationships in families
	4 Implications of heredity
	5 Juggling self‐care, caregiving and other responsibilities
	6 Facing life and future with HD
	7 Changing options and choices regarding education and employment
C Outside world	8 Communication about HD outside the family: What (not) to tell?
	9 Challenges to and struggles with social life and relationships
	10 Meeting the diverse needs of emotional, social and practical support
	11 Overcoming barriers accessing appropriate HD health services
	12 Societal lack of awareness and understanding

Each successive dimension encompasses a larger area of the individual's environment. While the first dimension relates strictly to the individual with HD, the second dimension includes the household of an HD family, and the third dimension includes friends, relatives, and society.

The following sections present the thematic loadings for each dimension, illustrating the lived experiences of the three family perspectives. The studies that reflect these experiences are outlined in Tables 4, 5, 6. To improve readability, references are not included in the text for experiences that can be derived from the tables. However, where the information is not available in the tables, it is referenced in the text.

### 3.3 Dimension A. Having the Disease

The dimension *Having the Disease* provides an overview of the physical and cognitive symptoms, behavioural changes, and disease progression related to HD. The experiences of individuals with HD, their informal caregivers, and offspring in this dimension are listed in Table 4.

**TABLE 4:** The experiences of dimension A. Having the disease.

Theme	The individual with HD shows	Reported in study (as numbered in Table 2)	Experienced by		
			I	C	O
Navigating the diverse symptoms	Physical symptoms				
	Deterioration in speaking ability	5, 12, 14, 15, 28, 35	◘		
	Deterioration in mobility (chorea, balance)	5, 6, 15, 27, 28, 30	◘	◘	◘
	Fatigue/loss of energy	1, 3, 14, 15, 35	◘		
	Sleeping disturbances	3, 5, 15, 31	◘		
	Decrease in swallowing	5, 15, 28	◘		
	Weight loss	5, 15, 27	◘	◘	
	Cognitive symptoms				
	Apathy (deterioration in initiation of activities and identifying goals)	1, 3, 5, 6, 14, 15, 29	◘	◘	
	Deterioration in language construction and comprehension	3, 5, 12, 14, 15, 35	◘	◘	
	Deterioration in learning and memory	3, 5, 12, 14, 15, 35	◘	◘	
	Deterioration in attention and concentration	3, 5, 12, 14, 15	◘		
	Lack of disease insight	5, 6, 19, 28, 33		◘	◘
	Deterioration of executive functioning (planning, organising, decision‐making, problem‐solving)	3, 5, 15, 33	◘	◘	
	Deterioration in processing information	5, 12, 15	◘		
	Behaviour and psychological symptoms				
	Aggressive and uncontrolled behaviour	3, 5–7, 14, 16, 22–24, 28–30, 32	◘	◘	◘
	Mood swings	6, 15, 33–35	◘	◘	
	Depression or depressive feelings	3, 5, 15, 28, 29	◘	◘	
	Changing personality (less likeable)	6, 14, 23, 28, 29	◘	◘	◘
	Anxiety	3, 5, 15	◘	◘	
	Deterioration of self‐confidence	14, 15	◘		
	Obsessional behaviour	6, 31		◘	
	Paranoia	29	◘		
Fading capacities: the toll of disease progression	Loss of independence or autonomy	3, 5, 15, 16, 21, 24, 26–29, 32, 34	◘	◘	◘
	Loss of identity	3, 29	◘		

Abbreviations: C, informal caregiver; I, individual with HD; O, offspring.

#### 3.3.1 Navigating the Diverse Symptoms

This theme demonstrates the considerable variability in the symptoms and signs of HD. Physical and cognitive symptoms are most frequently reported by individuals with HD, including apathy and deterioration in speech, mobility, learning and memory. In contrast, behavioural changes, particularly aggressive and uncontrolled behaviour, are predominantly reported by informal caregivers and offspring. When individuals with HD do mention such behaviour, it is typically in reference to past experiences of aggression from their parent who had HD. Given the prominence of these behavioural concerns among caregivers, Hubcikova et al. (2022) highlight that managing the mental symptoms of affected family members is more challenging than addressing motoric symptoms. The adjustments made by offspring and caregivers primarily revolve around managing these behavioural changes (Dawson et al. 2004; Rothing et al. 2014, 2015; Sparbel et al. 2008; Williams, Ayres, et al. 2009; Williams, Skirton, et al. 2009). Teenagers express concern about the unpredictability of the affected individual's behaviour, leading to daily uncertainty about whether it will be a ‘good’ or ‘bad’ day (Sparbel et al. 2008). They adapt their behaviour to prevent temper outbursts or undesired actions from their parent with HD. Additionally, partners are hesitant to leave children alone with the affected parent due to concerns about uncontrolled behaviour (Rothing et al. 2014). *‘He was getting very aggressive like we were walking on eggshells towards the end. He was starting to get aggressive and hitting. Most other things I could put up with, but he was hitting into the children.’* (Dawson et al. 2004, 127).

#### 3.3.2 Fading Capacities: The Toll of Disease Progression

As HD progresses, individuals with HD experience a gradual decline in function and activity, which increases the burden on informal caregivers (Rothing et al. 2015). In response to this decline, individuals with HD often prefer to be surrounded by people who knew them before the onset of HD, reflecting their desire for familiarity and understanding as they face behavioural changes (Soltysiak et al. 2008). Additionally, among the various challenges associated with disease progression, the loss of driving privileges is frequently cited as a significant loss of independence (Carlozzi and Tulsky 2013; Ho and Hocaoglu 2011; Sherman et al. 2020; Smith et al. 2015; Williams, Ayres, et al. 2009; Williams, Skirton, et al. 2009). *‘I lost my license this Autumn. (Before) I could just pop in my car and it would be ok, but now I have to depend on people, but I'm not used to that. It's been tough’* (Sherman et al. 2020, 6). While individuals with HD perceive it as a major loss, informal caregivers tend to find it less important (Smith et al. 2015).

### 3.4 Dimension B. Family Dynamics

The dimension *Family Dynamics* represents the experiences of living in a household affected by HD as an individual with HD, their informal caregiver and offspring. Table 5 shows what experiences of this dimension were reported in the included studies and who had these experiences.

**TABLE 5:** The experiences of dimension B. Family dynamics.

Themes	The I, C or O experiences	Reported in study (as numbered in Table 2)	Experienced by		
			I	C	O
Hidden conversations and strained relationships in families	Feelings of isolation in dealing with HD	2, 3, 6, 7, 11, 14, 19, 22, 24, 25, 30–33	◘	◘	◘
	Conflicting needs in communication within family	6, 8, 16, 18, 19–23, 25, 31, 34, 35		◘	◘
	Changing (intimate) partner relationship	2, 3, 6, 15, 23, 24, 26, 27, 31, 33, 34	◘	◘	
	Difficulties in relinquishing care due to strong sense of responsibility	2, 6, 7, 24–26, 30, 32–34		◘	◘
	Depression or depressive feelings	2, 3, 5, 11, 15, 16, 29, 30, 32	◘	◘	◘
	Changing parent–child relationship	6, 19–23, 30, 33		◘	◘
	Frustrations with secrecy in family	2, 11, 16, 17, 20, 21, 26		◘	◘
	Feelings of guilt due to (in)dependence	2, 5, 24, 25, 31, 34	◘	◘	
	Strengthened family ties due to HD	2, 3, 8, 23, 24	◘	◘	◘
	(Difficulties in) empathy for individual with HD	8, 23, 29, 32, 33		◘	◘
	Being at risk of physical/emotional harm	3, 11, 20, 32			◘
	Absence of non‐affected parent	11, 19, 30, 32			◘
	Difficulties in dealing with the confrontation of being diagnosed	7, 10, 21, 23	◘	◘	◘
	Walking on eggshells to prevent temper outbursts of individual with HD	7, 25, 30, 32			◘
	Loss of sharing meaningful communication or moments with individual with HD	14, 30, 31, 32		◘	◘
	Different caregiving roles among siblings	8, 21			◘
	Feeling forced to confront individual with HD about their limitations	31, 34		◘	
	Lack of empathy from individual with HD	33, 34		◘	
	Conflicts between family members	24, 30			◘
Implications of heredity	Different considerations when deciding if and when to test	5, 6, 8, 11, 16, 21–23, 29–31	◘	◘	◘
	Worries and daily reminders of own risk	3, 5, 7, 8, 11, 22, 23, 30, 32	◘		◘
	Trauma of caring for and losing loved ones to HD	3, 11, 13, 29, 31, 32	◘	◘	◘
	Doubts about whether it is right to have children	5, 6, 23, 30, 34	◘	◘	◘
	Feeling guilty about inheritance	3, 5, 16, 34	◘	◘	
	Being emotionally affected by the test results of family members	7, 8, 22		◘	◘
	Feeling misunderstood by the non‐affected parent in genetic risk	30			◘
	Not blaming parents for being at risk	8			◘
Juggling self‐care, caregiving and other responsibilities	Neglecting own needs and health	2, 6, 7, 11, 17, 19, 24–26, 28, 29, 31–34		◘	◘
	Need for respite facilities	2, 6, 7, 25, 28, 29, 33, 34		◘	
	Neglecting care for others besides the individual with HD	2, 11, 16, 23, 24, 31, 34		◘	◘
	Parentification	11, 16, 22, 24, 30, 32			◘
	Having difficulties completing other tasks besides caregiving	2, 17, 22, 24		◘	◘
Facing life and future	Uncertainty and anxiety about the future	2, 3, 5–9, 11, 13, 15, 22, 23, 26, 29, 31–34	◘	◘	◘
	Uncertainty due to unpredictability of ongoing progression of the disease	3, 5, 6, 19, 20, 23, 30–34	◘	◘	◘
	(Should need to start) thinking about end of life and future care	4, 5, 9, 13, 18, 21, 26, 28, 31	◘	◘	◘
	Need for daily structure and planning activities	1, 6, 7, 27, 28, 31, 34	◘	◘	
	Feeling constant presence of HD	3, 13, 17, 21, 23, 26, 30, 32, 33, 34	◘	◘	◘
	Sense of duty in caregiving	2, 8, 24, 26		◘	◘
	Thoughts of suicide of individual with HD	3, 5, 13, 24	◘	◘	
	Caregiving gives positivity	8, 26, 34		◘	◘
	Continuous search in coping with progression of HD	6, 25		◘	
	HD having an impact on future plans and activities in present	30, 32			◘
	Coping strategies				
	Ignoring or avoiding the presence of HD (*not talking about HD, avoiding crowded situations and other individuals with HD, creating own safe space in head or home*)	3, 5, 9, 11, 17–23, 25, 26, 29, 32, 35	◘	◘	◘
	Accepting the existence of HD (*gaining knowledge about family history, planning the future, regulation of information, assimilation, adapting, communicating open about HD, anticipatory grieving*)	1, 2, 4, 5, 6, 9, 11, 17, 23, 25–27, 29, 34	◘	◘	◘
	Having a positive attitude (*being grateful, using humour, having faith, being resilient, hoping for a mild deterioration, hoping for a cure and having faith in scientific research*)	2, 5, 7, 9, 11, 13, 17, 23, 26–28, 34	◘	◘	◘
	Trying to maintain a normal life (*maintaining familiar activities*)	7, 9, 11, 17, 25, 27, 28	◘	◘	◘
	Being watchful to progression of HD	5, 21, 27, 29, 34, 35	◘	◘	
	Living in the present (*one day at a time*)	4, 5, 7, 8, 9, 21	◘	◘	◘
	Getting treatment or medication to cope with HD in family	2, 11, 26, 34		◘	◘
Changing options and choices regarding education and employment	Worries about financial situation	2, 5, 7, 16, 24, 26, 30, 32–34		◘	◘
	Impact of HD symptoms on employment	3, 5, 16, 28	◘		
	Giving low priority to school	5, 11, 25, 30	◘	◘	◘
	Making adjustments in career/work	26, 34		◘	
	Working provides a break from caring	25, 31		◘	
	Rarely discussing college/career plans	30			◘

Abbreviations: C, informal caregiver; I, individual with HD; O, offspring.

#### 3.4.1 Hidden Conversations and Strained Relationships in Families

The experience most mentioned in the literature on this theme is a sense of isolation, that affects all family members. These feelings arise from changes in relationships, ongoing losses, and, for the offspring, the added burden of being at risk of developing HD. The lack of knowledge and understanding from friends and relatives exacerbates these feelings of isolation (Bilal et al. 2022; Daemen et al. 2023; Dondanville et al. 2019; Grimstvedt et al. 2021; Ho and Hocaoglu 2011; Kavanaugh et al. 2015; Kjoelaas, Feragen, et al. 2022; Kjoelaas, Jensen, et al. 2022; Smith et al. 2015; Sparbel et al. 2008; Williams et al. 2012; Williams, Ayres, et al. 2009; Williams, Skirton, et al. 2009). *‘I recognize the loneliness that [other caregiver] is talking about. The entire process takes a long time, and you don't want to burden others with your grief all the time. Consequently, you postpone sharing your feelings.’* (Daemen et al. 2023, 4).

Due to the heritable nature of HD, families have to deal with a sense of loss caused by their current situation while simultaneously experiencing future‐oriented loss (Aubeeluck et al. 2012).

Feelings of responsibility that make it impossible to leave the individual with HD alone are present in informal caregivers and in offspring who take on a caring role. This makes it difficult to meet their own needs for social activities and self‐care. *‘My main concern probably is like, when I go off to college, how's my mom going to get around, because when I graduate, my dad's planning on moving back east, so I don't know how she will get her groceries.’* (Williams, Ayres, et al. 2009).

Discussing HD with offspring is a complex task, with many families lacking guidance from health services (Kjoelaas, Feragen, et al. 2022; Kjoelaas, Jensen, et al. 2022). Dilemmas arise, such as offspring wanting to talk and gather information about HD as early as possible, while the affected parent may deny the disease to protect the child, maintain normalcy or out of fear of compromising the bond (Kjoelaas, Feragen, et al. 2022; Kjoelaas, Jensen, et al. 2022). Delaying disclosure until adolescence can lead to feelings of deception in the offspring, creating a barrier to trust between them and their parents (Kjoelaas, Feragen, et al. 2022; Kjoelaas, Jensen, et al. 2022). Growing up with knowledge about HD seems to facilitate better coping mechanisms for the offspring (Forrest Keenan et al. 2007). Informal caregivers are often caught between these conflicting needs, balancing the needs of the offspring with those of the individual with HD (Kjoelaas, Feragen, et al. 2022; Kjoelaas, Jensen, et al. 2022). *‘I knew for years that she [partner with HD] would get the disease, but I couldn't tell the kids because that would go against what she wanted. Of course, I could have done that, but I didn't feel it was right*.*’* (Kjoelaas, Feragen, et al. 2022; Kjoelaas, Jensen, et al. 2022). Parent–child relationships may also be negatively affected by the loss of communication skills of the individual with HD, feelings of misunderstanding about being at risk by the non‐affected parent, the absence of the non‐affected parent, and the possible caring role that the offspring may take on.

Some families experience the presence of HD as a source of family strength. Maxted et al. (2014) explore how families function as closed units, providing mutual support and protection. *‘If we deny that the disease exists what good will that do? We have to always remain strong and open with our kids so that they know we love them and support them and as a family we can get through whatever life's challenges are. HD has brought us closer.’* (Aubeeluck et al. 2012, 1432). In some cases, family members resist being part of this closed unit, withdrawing or feeling trapped when others try very hard to involve them (Maxted et al. 2014). ‘*So she sort of dismisses it all really. I've tried kind of getting it out of her but to be honest, […] I just can't be bothered to keep trying with her,’* ‘*cos, you know, she will come to us eventually when she needs that support. […] she can keep it all inside as long as possible, but she will be the one going through the depression and she'll be the one feeling isolated and, you know, and we'll just be there to pick up those pieces I think*.’ (Maxted et al. 2014, 344).

#### 3.4.2 Implications of Heredity

Young adults face complex decisions about genetic testing for various reasons. Some choose to test to prepare for the future, address insurance concerns, plan for children or relationships, or reduce feelings of uncertainty and anxiety. Conversely, others choose not to test due to fear of limiting future options, fear of reacting to test results and hurting family members, and the knowledge that testing will not change their genetic status or prevent disease manifestation. The timing of testing is also crucial. *‘Part of the reason I've been waiting to test is I don't necessarily know if I would want my dad or siblings to know the result because I think that'd be harder on them. […] I feel like it would crush them, maybe even more than it would crush me, if it was positive.’* (Dondanville et al. 2019, 297).

Forrest Keenan et al. (2007) highlight the detrimental impact of being at risk, which can manifest physically (e.g., fatigue), emotionally (e.g., reduced self‐esteem, avoidance of relationships) and psychologically (e.g., being watchful for symptoms, severe anxiety, insomnia). The psychological impact of being constantly surrounded by, and possibly getting, HD takes up space in a teenager's emotional life (Williams, Ayres, et al. 2009). *‘When this is over, and if you don't get it, you'll be a better person in the long haul. You'll have, uh, spent your time in hell, so … if you don't get this … then, your life's going to be great because you went through hell already.’* (Williams, Ayres, et al. 2009).

Additionally, Dondanville et al. (2019) found that siblings caring for a parent with HD were more willing to undergo testing compared to non‐caring siblings. The family taboo surrounding discussions about testing further complicates decision‐making. *‘I think that does have an impact on why she doesn't want to get tested because she has tried to block out the caregiving, and so now she's trying to block out whether or not this is going to happen to her as well. So if I didn't have to care for my mom then honestly, I don't know if I would get tested because it's like I'm not watching someone decline from it.’* (Dondanville et al. 2019, 297).

#### 3.4.3 Juggling Self‐Care, Caregiving and Other Responsibilities

Daemen et al. (2023) introduce the ‘*care paradox*’, emphasising that self‐care and maintaining one's own valued activities are essential to continuing to care for a loved one with HD. Unfortunately, as the disease progresses, the act of caring tends to overshadow the caregiver's personal needs and ability to live their own life (Rothing et al. 2014). The needs of other family members may also be neglected. Fulfilling responsibilities, such as working or going to school, raising children and doing household chores, is put at risk. Routine household tasks may become a barrier to the caregivers' caring responsibilities. In families that are under such pressure, even minor stressors could result in crises (Aubeeluck et al. 2012). ‘*You are dependent on everything running ok and it only takes one tiny thing to make everything fall apart. It's like being on a short fuse.’* (Aubeeluck et al. 2012, 1431).

Offspring often take on adult‐like care responsibilities, including dressing, feeding and coordinating care, known as parentification. Despite their important role, they lack legal decision‐making authority (Williams, Ayres, et al. 2009; Scerri 2015). Having responsibilities without the ability to make changes makes this a very demanding role. Their caring experiences were characterised by emotional distress, responsibility and reduced social time. Forrest Keenan et al. (2007) describe caring young adults juggling multiple responsibilities as *‘the linchpin of family systems’*. *‘Before school we had to get my mum bathed, changed, get her breakfast, plus get us showered and changed. Plus, you'd have to do all the tidying up before you went. And then coming home tea would have to get made. I missed most of my third year at school because of it. I didn't like leaving mum here on her own during the day while me and my sister were at school and my dad was out working so some days, I would skive off just to make sure she was all right.’* (Forrest Keenan et al. 2007, 122).

#### 3.4.4 Facing Life and Future

All three perspectives voice concerns about the future. Mostly about the future of their offspring, whether they will get the disease, but also about the uncertain course of the disease. Due to the diversity and variability of the symptoms, the progression of the disease cannot be anticipated. This course involves unpredictable, ongoing experiences of change and loss (Velissaris et al. 2021). *‘You never know what the next thing's gonna be… and it's not something that anybody can tell you, and it's not something that [you know] if you're gonna be able to slow down, or if you're gonna be able to fix…’* (Sparbel et al. 2008, 5).

The constant presence of HD in the family and waiting for the onset and progression of the disease, as seen by other family members in the past, is described by Maxted et al. (2014) as *‘a spectre hanging over us.’ ‘it feels like there is no place to go where I can express the pain involved in this and the guilt because there are times I feel as though I can't take it anymore. It is often a painful place mixed with cries of despair and then times when all goes as well as can be expected but it seems lately I am running out of the heart to go on being the one responsible for mum's needs while feeling the disease myself but there is no‐one else to help, it is my job.’* (Aubeeluck et al. 2012, 1429).

##### 3.4.4.1 Coping Strategies

Family members use different coping strategies depending on the stage and progression of the disease (Rothing et al. 2015) and the stage of family life when the disease occurs (Rothing et al. 2014). Overall coping strategies are illustrated, accompanied by examples, in Table 5. Using several strategies at the same time is common among individuals with HD (Ekkel et al. 2021). All three perspectives often cope by ignoring the presence of HD by withholding information about inheritance to reduce stress, not fully considering the long‐term consequences for their children, and avoiding thoughts of future deterioration. *‘No, but obviously I'm sort of burying my head in the sand a little, [laughs], [*…*]. Of course that helps you to keep going, yes. [*…*] Often just not think about it, about what it will be like in 5, 6, 7, 8 years from now. Easier*.’ (Ekkel et al. 2021, 5). Taking it one day at a time is also a frequently used coping strategy, often combined with ignoring the disease. *‘You can't really plan for it—I just take 1 day at a time. It's so slow you just adapt. I don't make plans.’* (Lowit and van Teijlingen 2005, 5). Being watchful, i.e., wondering if every change in condition signals the progression of HD, and avoiding the existence of HD presents contradictory strategies (Lowit and van Teijlingen 2005). '*When I spotted the first signs I just kind of hoped that it wasn't it. I suppose I knew it was, but still I didn't think about it at the time.* (van Teijlingen 2005, 5).

For the offspring, the complexity of being at risk and possibly being in a caring role makes it a particularly challenging situation and may lead to siblings adopting conflicting coping strategies. *‘I see everybody else as different and my mum's normal because I've lived with it, whereas everybody else would see my mum as different but I don't. My sister gets upset about it quite a lot of the time. She would rather hide in a corner and cry about it. She doesn't find it as easy as me. I'll walk down the street with mum and not care what anybody else thinks but she won't.’* (Forrest Keenan et al. 2007, 125).

#### 3.4.5 Changing Options and Choices Regarding Education and Employment

All three perspectives experience an impact of HD on education and employment, often leading to financial worries. Individuals with HD experience changes in work routines and difficulties at work due to HD symptoms. *‘I haven't been able to find a job for a long time, I act like I'm an alcoholic with my involuntary movements. And once I got a job, I almost everywhere had an accident at work.’* (Hubcikova et al. 2022, 9). Informal caregivers adjust their work routines to allow flexibility for caring, while offspring carefully consider educational choices or deprioritize school, something they might not have done if the family was not affected by HD.

### 3.5 Dimension C. Outside World

The dimension *Outside World* presents an overview of the experiences of relationships and interactions with relatives, friends, health services and society. All experiences of the individuals with HD, their informal caregivers and offspring of this dimension are shown in Table 6.

**TABLE 6:** The experiences of dimension C. Outside world.

Theme	The I, C or O experiences	Reported in study (as numbered in Table 2)	Experienced by		
			I	C	O
Communication outside the family: What (not) to tell?	Dilemma between respecting the other family members' autonomy and receiving support	2, 20, 24, 25		◘	◘
	Openness can lead to (mis)understanding	6, 20, 22, 35	◘	◘	◘
	Differences in need to talk about the family situation outside the family	19–22		◘	◘
	Caution about who to tell about HD	20, 22, 25	◘	◘	◘
Challenges to and struggles with social life and relationships	Need for social support	1–3, 7, 8, 11, 14–17, 19, 22, 24, 26, 28, 29, 33, 35	◘	◘	◘
	Declining social life: losing and avoiding friendships and social activities	3, 5, 6, 14, 15, 17, 21, 24, 26, 27, 29, 32–34	◘	◘	◘
	Receiving no/minimal understanding of others (outside the family)	3, 6, 8, 12, 15, 17, 19, 28, 30, 33, 34		◘	◘
	Having conflicts or complex relationships with relatives	14, 15, 19, 21, 22, 24, 30, 31, 33	◘	◘	◘
	Avoiding contact between friends and affected parent	20, 30, 32			◘
Meeting the diverse needs of emotional, social and practical support	(Conflicting) needs regarding peer support	2, 5–7, 15, 9, 17, 19, 21, 28–31	◘	◘	◘
	Need for emotional and psychological support	3, 7, 10, 11, 15, 17, 19, 20, 26, 28	◘	◘	◘
	Need for practical support	6, 7, 15, 17, 19, 26, 28	◘	◘	◘
	Need for information about clinical trails	16, 27, 28	◘	◘	
	Not wanting involvement from outsiders	2		◘	
Overcoming barriers accessing appropriate health services	Lack of regulated, customised information and flexible care	1, 5–7, 10, 11, 16, 25, 28, 29, 31	◘	◘	◘
	Lack of empathy and understanding by professionals	2, 7, 11, 16, 21, 26, 28, 29	◘	◘	◘
	Lack of knowledge about HD in (primary) care	2, 6, 7, 10, 16, 28, 29	◘	◘	
	Lack of continuity in care	6, 7, 10, 28, 29	◘	◘	
	Lack of, and difficulties in, accessing information and education	2, 7, 16, 17, 28		◘	◘
	Difficulties in or need for accessing the specialist services	2, 10, 28, 29	◘	◘	
	Worries about availability and accessibility of future care	7, 10, 30	◘	◘	◘
	Wanting faster access to research drug	28	◘		
Societal lack of awareness and understanding	Stigma and misconception in society about HD	5, 6, 15, 27, 29	◘	◘	◘
	Legal constraints to coordinating the care required	26, 32		◘	◘
	Practical difficulties in accessing public spaces	26		◘	

Abbreviations: C, informal caregiver; I, individual with HD; O, offspring.

#### 3.5.1 Communication Outside the Family: What (Not) to Tell?

Within families, communication dilemmas arise regarding openness versus secrecy about HD (see Table 5). The persistence of these dilemmas beyond the family context are shown in Table 6. Questions arise about who is entitled to share information with whom, how much should be communicated to the outside world, and what the potential consequences for family members might be. The balance between protection and disclosure is crucial (Rothing et al. 2015). Discussing HD openly can promote understanding and support, but it can also lead to misunderstandings, affect the autonomy of the individual with HD, or interfere with the evolving needs of offspring as they grow older (Kjoelaas, Jensen, et al. 2022). Some offspring experience that they want to share information about their situation but have been hindered by their parents' desire to maintain secrecy. Family members approach this communication with caution (Kjoelaas, Jensen, et al. 2022). *‘I have chosen to be open about it at work, but not with my affected husband's family. His daughter's parents‐in‐law can see that something is wrong, but we are not allowed to talk about the reason why. His daughter has been tested, and has her own children. They want to protect her husband's family from knowing about HD.’* (Rothing et al. 2015, 572).

#### 3.5.2 Challenges and Struggles in Social Life and Relationships

The decline in social life of individuals with HD and their informal caregivers is caused by avoidance of others and crowded situations, chorea, less contact with friends due to changes in the individual with HD, strains in relationships, and misunderstanding by friends and relatives. *‘Other people don't understand—it's much easier to kind of keep it to yourself and not share with people. Because, when you do, they look at you a bit askance, like you're imagining things.’* (Williams, Ayres, et al. 2009; Williams, Skirton, et al. 2009).

Some offspring feel unable to fully open up to their friends; they feel misunderstood and avoid contact between their friends and the affected parent. Due to caregiving responsibilities and managing the challenging behaviour of the parent with HD, their time spent with friends becomes restricted (Williams, Skirton, et al. 2009, 7).

#### 3.5.3 Meeting the Diverse Needs of Emotional, Social and Practical Support

The need for various forms of support from family, friends and health services is recognised from all three perspectives. This support includes practical advice, such as dietary recommendations; emotional support, including empathy from friends; psychological support such as managing anxiety and guidance on disclosing the disease to offspring; and the availability of clear educational information, for example, to help people to better understand the stages of HD. There is a delicate balance between the level, amount and timing of information and support (Dawson et al. 2004), and needs may vary and conflict between family members. *‘It's the most difficult part about this whole thing when you are a young carer who wants help, but you are not getting anywhere because your parent is denying that they have a disease’* (Kjoelaas, Feragen, et al. 2022, 665). Despite their dedication, many caregivers felt unsupported by health services and described their experiences to getting adequate support as a constant struggle (Soltysiak et al. 2008).

Young caregivers most in need of additional support were often from families where the non‐affected parent worked long hours, was also ill, or where the affected parent was a single parent (Forrest Keenan et al. 2007). Because of this lack of parental presence, children have to rely more on social support outside the parent–child relationship (Kjoelaas, Feragen, et al. 2022). Forrest Keenan et al. (2007) highlight the importance of social support systems and trusted relationships as protective factors for offspring to manage their family situation and personal risk effectively.

Peer support plays a dual role in coping with HD. It can be a barrier due to fears of confronting the disease's future impact and reluctance to engage with other people's struggles. However, it also acts as a source of resilience, alleviating isolation and providing practical guidance. *‘We actually get more information out of the support group because there are people in different stages of Huntington's. That's more useful for us and it's actually since he's been involved with the support group, he's much happier because he can see other people in his situation and you don't have to be depressed about it.’* (Dawson et al. 2004, 127).

#### 3.5.4 Overcoming Barriers Accessing Appropriate Health Services

The need for customised flexible care and information is mentioned by all three perspectives. When healthcare services fail to meet these needs, informal caregivers often experience frustration, anger, and despair (Aubeeluck et al. 2012). *Some people want information and some people don't…some people want to talk about Huntington's and other people don't. You know, it's a very intuitive, personal thing* (Soltysiak et al. 2008, 232).

While most experiences were reported by individuals with HD and their informal caregivers, offspring also face difficulties in finding accurate information. All three perspectives emphasise the importance of regulation, which means avoiding an information overload and allowing choice in receiving disease related details. *‘I don't really know that much about it, but I know enough. I'm ok, I don't know if I would really like to find out anymore, I'm happy with what I know just now. I think if mum was getting worse and worse, I think I would like to know more.’* (Forrest Keenan et al. 2007, 125).

#### 3.5.5 Societal Lack of Awareness and Understanding

The fact that other people have no idea of what the condition entails leads to a great deal of misunderstanding experienced from all three perspectives. These misunderstandings can lead to preconceptions and stigmatisation of the disease. *‘They always thought that I was drinking… Eleven years of pee tests, every cop, every person in the town that I came from thought I was a drunk.’* (Sherman et al. 2020, 6).

## 4 Discussion

This scoping review aimed to provide an integrated view and a holistic understanding of the multifaceted challenges faced by families affected by HD. We mapped and integrated available literature on the lived experiences of daily living with HD from three perspectives: individuals with HD, their informal caregivers, and their offspring. To provide a structured overview, we clustered these lived experiences into 12 themes, grouped into three dimensions.

The first dimension, *Having the Disease*, shows that individuals with HD primarily report experiences related to their physical and cognitive symptoms, whereas informal caregivers and offspring emphasise behavioural changes. This difference in perspectives is confirmed in one of the included studies by Carlozzi et al. (Carlozzi and Tulsky 2013), who conducted focus groups with individuals with HD, informal caregivers, and healthcare providers. Behavioural changes and psychiatric symptoms were notably absent in the reports of individuals with HD, but were frequently discussed by the other two groups. The authors attributed this to a lack of insight into the disease, which our review highlights as a burdensome symptom for informal caregivers and offspring. Indeed, a lack of insight into the disease in individuals with HD can lead to overestimation of their own abilities, resulting in improper medication use, missed clinic appointments, and underreporting of daily difficulties, thereby exacerbating the burden on informal caregivers (Wibawa et al. 2020). This burden is substantial and increases over time, particularly for irritability and obsessive‐compulsive behaviours in individuals with HD compared to those with apathetic symptoms (Youssov et al. 2022). De Carolis et al. (2015) further illustrate this point by showing that more severe psychiatric symptoms are associated with less awareness of cognitive deficits. The interplay between behavioural changes and lack of insight into the disease significantly amplifies the burden on informal caregivers and offspring. Studies have reported similar findings in relation to the burden experienced by informal caregivers of individuals with dementia. In dementia, disruptive behaviours like agitation, aggression, and severe anxiety are often the most challenging to manage and significantly hinder the ability to care for individuals with dementia (Mendez 2022; Turró‐Garriga et al. 2013). The lack of insight into the disease in individuals with dementia also exacerbates these challenges and increases the burden on informal caregivers (Turró‐Garriga et al. 2013).

The aspects that impact on a family with HD are further explored in the second dimension, *Family Dynamics*, which summarises the experiences of living in a household affected by HD. Our review suggests that the ongoing burden on informal caregivers and offspring, combined with the divergent needs within a family, could explain the vital need for personal space and an independent life without having to deal with the disease. This, however, often feels impossible for family members. The lack of personal space causes emotional distress in informal caregivers and may ultimately reduce their life expectancy (Schulz et al. 1995). This emotional distress and its associated vulnerability to mental health problems are also experienced by the offspring (Cooper et al. 2025; Tuck et al. 2023). Exuzides et al. (2022) show that mental health comorbidity is nearly twice as prevalent in HD families compared to those affected by Parkinson's disease, underscoring the profound mental impact of informal caregiving in HD. The sense of isolation, experienced by all three perspectives, reflects the mental burden that HD imposes on families. In addition, the complex family dynamics that are specific to HD, can have a reciprocal effect on all family members' mental health (Tuck et al. 2023). This underlines the urgent need for adequate mental health and preventive services tailored to HD families (Exuzides et al. 2022). Thereby, the burden on informal caregivers and offspring is further exacerbated by the genetic inheritance of the disease (Parekh et al. 2018). This introduces unique challenges, such as transmission guilt, worries about the offspring's future, and clinical depression among informal caregivers and offspring (Sandilands et al. 2022). These additional challenges contribute to the particularly complex and conflicting needs within families affected by HD, making their experiences distinct from families dealing with other neurodegenerative diseases (Youssov et al. 2022).

One of the most common conflicting needs among family members is the need for communication and secrecy around HD. Secrecy within families often strains relationships, as parents may limit communication to protect their children, while offspring initially find it difficult to bear. Offspring appreciate honest and transparent information, which enables them to discuss, share experiences, and manage living in a family affected by HD. Families that maintain open communication report that children are more emotionally and psychologically resilient, often responding pragmatically to their genetic risks (Metcalfe et al. 2008).

While open communication within the family is crucial for emotional resilience, openness beyond the family circle can also have significant benefits. Being open about HD outside the family, for instance, can foster appropriate support and help reduce the stigma surrounding the disease. The third dimension of our review, *Outside World*, shows that all three perspectives experience high levels of misunderstanding from friends, relatives, and health services. HD has been recognised as one of the most stigmatised disorders, leading to widespread misconceptions and misinformation about the behaviours and needs of individuals with HD (Edge 2010). This stigma not only shapes public attitudes but also fosters genetic discrimination, particularly in contexts such as employment and health insurance (Edge 2010). Fearing this discrimination, individuals with HD and their families often adopt coping mechanisms of secrecy or denial (Wauters and Van Hoyweghen 2021). This public stigma and associated genetic discrimination can lead to self‐stigma which further isolates individuals and reinforces the cycle of stigma (Corrigan and Watson 2002). Self‐stigma manifests when individuals internalise the public stereotypes and prejudices associated with their condition, leading to feelings of shame, diminished self‐worth, and a reluctance to seek support or disclose their condition (Corrigan and Watson 2002). The fear of being treated unfairly due to one's genetic status intensifies the urge to hide the disease, which in turn perpetuates public misconceptions and the stigma surrounding HD. This vicious cycle conflicts with the urgent need for adequate support and recognition of the disease within HD families. As our review suggests, the perceived lack of support is related to a reduced social life, avoidance of others, misunderstandings, conflicts with relatives, and feelings of isolation. These experiences appear to be mutually reinforcing. Although adequate support is needed, many caregivers avoid peer group meetings because of the vision of the future that is presented, whereas informal caregivers of other neurodegenerative diseases seek this support and want to know their vision of the future (Lowit and van Teijlingen 2005). Lowit and van Teijlingen (2005) attribute this to the hereditary nature of HD: it may also reflect the future vision of other family members. Conversely, when families receive adequate social support, it significantly improves their quality of life and well‐being (Mo et al. 2022).

**FIGURE 2:**
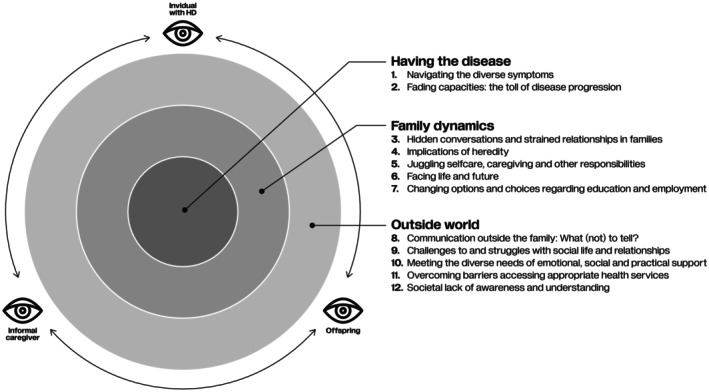
Intertwined themes and dimensions and shifting perspectives.

The collated findings of all the studies included in our review show a complex interplay of experiences within and surrounding families affected by HD. This complexity is further compounded by the different and often conflicting needs and coping strategies of family members. The experiences of family members are entangled within and across dimensions and perspectives, underscoring the interconnectedness of the 12 themes (see Figure 2). Additionally, due to the nature of HD as a family disease, the boundaries between roles and perspectives may shift or fade: individuals with HD may have childhood or caregiver experiences with their own affected parent, and children may eventually become informal caregivers or develop HD themselves.

### 4.1 Implications for Practice

This review provides a comprehensive overview of experiences from the three perspectives which can serve as a practice tool for healthcare professionals. This tool can help them to identify the diverse experiences and needs of family members within a family affected by HD, thereby offering deeper insights into the familial dynamics and challenges they face.

Second, given the hereditary and progressive nature of HD and the complexity within HD‐affected families, it is recommended that healthcare professionals adopt a systemic family approach. Current care for individuals with HD primarily focuses on the individual with HD (Soltysiak et al. 2008), but this review emphasises the need for a holistic approach that addresses the needs of the entire family. The need for a family approach is also emphasised in the review by Cooper et al. (2025) based on experiences of growing up in a family affected by HD. Family involvement in the current treatment of HD may be underexposed; yet in mental health treatment, family involvement has been shown to lead to better outcomes with psychological therapies and pharmacological treatments, fewer hospital admissions, shorter hospital stays, and better quality of life (Eassom et al. 2014). These practices offer valuable insights for the treatment of HD, where systemic family approaches could similarly enhance care outcomes and family well‐being. Adopting such approaches in HD care could mitigate the emotional strain on families, provide more coordinated support, and reduce the isolation that many family members experience. A promising example from mental health care that may be appropriate for families affected by HD is the Family‐focused practice (FFP). FFP is specifically designed for families in which a parent has a mental illness and aims to reduce the significant risks associated with parental mental health problems. FFP focuses on (1) family planning and goal‐setting, (2) family and service linkages, (3) individual and family support, (4) individual and family assessment, (5) psychoeducation, and (6) a coordinated system of care between families and services (Tuck et al. 2023). This model could be adapted to the needs of HD families. Interventions that target the psychological well‐being of individuals with HD are also likely to have a positive impact on informal caregivers (Pickett et al. 2007; Scerri 2015), emphasising the interconnected nature of family health in HD care.

### 4.2 Limitations

A methodological limitation of this scoping review is that the screening of titles and abstracts was primarily conducted by the first author, which may have resulted in bias. To mitigate this, the second and third authors were involved in various stages of the data extraction and analysis, helping to ensure the trustworthiness of the review process.

The 35 articles showed variations in the way the lived experiences were described. Some articles discussed experiences in general terms, such as ‘cognitive problems’ or ‘psychological symptoms’, without providing specific details. These general experiences have been excluded from Tables 4, 5, 6.

The interconnectedness of the 12 themes allowed some lived experiences to be addressed under more than one theme. The authors jointly decided on the most logical theme to organise the experiences, aiming to stay as close as possible to the meaning of the source data. Additionally, the distinction between needs, barriers, facilitators, and experiences was sometimes ambiguous and interchangeable (e.g., a barrier could also reflect a need). In the tables, the needs of the population and the barriers experienced have been combined in an appropriate way.

The articles included are all from Western countries. As mentioned in the introduction, there are differences in the prevalence of HD worldwide. The experiences of families from non‐Western countries may be different due to different circumstances, such as the healthcare available.

Finally, in this review, we have focused on the most traditional family composition, where the individual with HD is the biological parent of the offspring, and the informal caregiver is typically the partner of this individual. However, other household configurations, such as siblings or extended family members acting as caregivers, may result in different experiences.

## 5 Conclusion

This scoping review provides a comprehensive overview of the intertwined common and distinct experiences and needs from three perspectives within families affected by HD, namely the individual with HD, their informal caregiver, and their offspring. The different needs within a family may be in conflict, complicating daily life and the provision of appropriate support. The burden of HD appears to differ from that of other neurodegenerative diseases due to its unique challenges, including the hereditary nature of the disease. Across all three perspectives, the psychological component significantly impacts daily life. It is recommended, therefore, that future interventions adopt systemic approaches from the mental health sector to address these complex familial needs.

## Author Contributions

Conceptualization; methodology and analysis; writing – original draft preparation; visualisation. Methodology and analysis; writing – review and editing. Writing – review and editing; methodology and analysis. Writing and editing. Supervision. All authors have agreed on the final version and meet at least one of the following criteria (recommended by the ICMJE (http://www.icmje.org/recommendations/)): (1) substantial contributions to conception and design, acquisition of data, or analysis and interpretation of data; (2) drafting the article or revising it critically for important intellectual content.

## Conflicts of Interest

The authors declare no conflicts of interest.

## Data Availability

Data sharing not applicable–no new data generated, or the article describes entirely theoretical research.

## Appendix

(“Huntington Disease”/OR (huntington*).ab,ti,kf.) AND (“Evaluation Study”.pt. OR “Evaluation Studies as Topic”/OR (facilitator* OR (barrier* NOT blood–brain‐barrier*) OR burden* OR (stress* NOT (oxidative‐stress* OR protein‐stress* OR protein‐in‐stress* OR ER‐stress*)) OR strain OR resilien* OR (needs NOT metabolic‐need*) OR self‐sustain* OR balance* OR demand* OR difficult* OR resource* OR benefit* OR support* OR strength*).ab,ti,kf. OR (evaluat* OR critiq* OR capacit* OR impact*).ti.) NOT (*“Metabolism”/OR *“Deep Brain Stimulation”/OR (metabol* OR deep‐brain* OR brain‐depth* OR drug‐intervent*).ti.) NOT (news OR congres* OR abstract* OR book* OR chapter* OR dissertation abstract*).pt.
